# Sociodemographic inequalities in non-small cell lung cancer (NSCLC) novel treatments: a national population-based cohort study

**DOI:** 10.1136/jech-2025-225545

**Published:** 2026-06-30

**Authors:** Sarah Rae, Erin Pennock, Adam Todd, Alastair Greystoke, Linda Sharp

**Affiliations:** 1 Newcastle University, Newcastle upon Tyne, UK; 2 Northern Centre for Cancer Care, Newcastle upon Tyne, UK; 3 Newcastle University Population Health Sciences Institute, Newcastle upon Tyne, UK; 4 Sir Bobby Robson Cancer Trials Research Centre, Northern Centre for Cancer Care, Newcastle upon Tyne, UK

**Keywords:** COHORT STUDIES, DRUG PRESCRIPTIONS, HEALTHCARE DISPARITIES, Health inequalities, NEOPLASMS

## Abstract

**Background:**

Sociodemographic inequalities impact patients with non-small cell lung cancer (NSCLC). Advances in novel therapeutics have increased treatment options; however, for advanced disease, prognosis remains poor and inequalities in care are recognised to exist, requiring further characterisation. This population-based study aimed to describe the associations between markers of sociodemographic characteristics and novel anticancer therapy utilisation for a stage IV NSCLC population in a publicly funded healthcare system.

**Methods:**

A retrospective cohort of 73 640 patients with histologically confirmed stage IV NSCLC between 1 January 2017 and 31 December 2021 was identified from the English national cancer registry and linked to the Systemic Anti-Cancer Therapy database. Multivariable logistic regression evaluated likelihood of novel therapy utilisation across sociodemographic characteristics and for pre-COVID-19 and peri-COVID-19 time periods.

**Results:**

Patients resident in the most deprived quintile had the lowest likelihood of novel therapy receipt. Patients ≥70 years old had reduced likelihood of receipt (OR: 0.79, CI 0.73 to 0.86), as did patients recorded as ethnicity ‘unknown’ (OR: 0.85, CI 0.75 to 0.97). Likelihood of utilisation increased from 2017 to 2019 and decreased in 2020 and 2021. Pre-COVID-19 and peri-COVID-19 analysis identified reduced sociodemographic inequalities from 2020, but variation across therapy subgroups.

**Conclusion:**

Even in the National Health Service (NHS) in England, where cancer medication access is free, inequalities in NSCLC novel therapies utilisation persisted in 2017–2021, despite more treatment options and greater clinician familiarity. There is evidence of decreasing utilisation during this study period. These findings are important to ensure equitable access to novel therapies and require further study of future trends and impact on real-world survival.

**WHAT IS ALREADY KNOWN ON THIS TOPIC**

- It is recognised that inequalities in cancer care affect patients with non-small cell lung cancer (NSCLC) throughout their cancer journey; however, the specific impact of sociodemographic factors on novel therapy utilisation is unknown.

**WHAT THIS STUDY ADDS**

- This study demonstrates sociodemographic inequalities in utilisation of novel therapies for NSCLC in the UK. Variation in utilisation was found between sociodemographic factors, drug types and in pre-COVID-19 and peri-COVID-19 time periods.

**HOW THIS STUDY MIGHT AFFECT RESEARCH, PRACTICE OR POLICY**

- As a disease of unmet need, in which sociodemographic inequalities in treatment have been demonstrated to exist, future work must focus on interventions to provide equitable access to care for patients with NSCLC.

## Introduction

 It is increasingly recognised that sociodemographic inequalities in cancer care affect patients throughout their diagnosis and treatment journey. Sociodemographic factors are characteristics constituting a combination of social and demographic factors such as age, sex, race, socioeconomic status and educational attainment, which are recognised to play an important role in health outcomes.^1^ Such inequalities are well characterised in non-small cell lung cancer (NSCLC) with variations between sociodemographic groups in experiencing later stage diagnosis^2^; delays in commencing definitive treatment, including surgery^3 4^; and, ultimately, differing survival.^5^ Despite improvements in lung cancer management, for patients diagnosed initially with stage IV disease, prognosis remains poor.^5^

Advances in novel systemic therapies over the previous decade have rapidly expanded the treatment options available. These novel anticancer therapies have included molecular targeted therapies based on tumour biomarker status; biological treatments with no predictive biomarker (eg, bevacizumab); and immune checkpoint inhibitors (ICIs) (eg, nivolumab). Newer therapies, including antibody-drug conjugates, are emerging which may further increase therapeutic options for people with metastatic disease.^6^ Such medications are instrumental in a shift to a more personalised approach to NSCLC management; this approach has been shown to improve patient outcomes.^7–9^

The inequalities in diagnosis and broader management of NSCLC have been shown to extend to utilisation of such novel cancer therapies. There is a growing body of real-world evidence recognising inequalities in access to novel therapeutics in cancer across sociodemographic characteristics.^10–12^ This includes work by Norris *et al* demonstrating inequalities relative to NSCLC patients’ socioeconomic status in England,^13^ a setting where care is free at the point of delivery. However, current data relates to when these treatments first started to become available—much less is known about practice and utilisation in more recent years.

As clinicians become increasingly familiar with such therapeutics, it might be expected that these inequalities may decrease. On the other hand, as therapeutics become more established, there is also a risk of such inequalities becoming embedded into practice.^14^ Furthermore, many of these therapies are targeted to specific mutations in the tumour: these can only be used if the mutations are tested for and identified. In the case of more recently significant mutations, identification of these has only recently become standard of care. The decision to test, and so be able to treat, remains impacted by clinician choice. While usage of such molecular testing has increased overall across previous years,^15 16^ this increase has not been equal across sociodemographic groups and risks accentuating inequalities in treatment receipt.^17–19^

Across this period of significant advancement in novel therapies, it is possible that prescribing practices have been altered by the COVID-19 pandemic. As a time of grave uncertainty, interim oncology treatments were introduced in the UK for NSCLC and other tumour sites, often to minimise patient time in hospital. In the context of novel therapies, examples included pembrolizumab single agent as first-line treatment for NSCLC and a programmed death ligand-1 (PDL-1) score of 1%–50% and dabrafenib plus trametinib for B-type rapidly accelerated fibrosarcoma (BRAF) positive metastatic disease instead of chemotherapy.^20^ While this should have opened access to novel therapies, it has been recognised that during the pandemic, fewer patients were diagnosed with, and commenced treatment for, lung cancer compared with previously.^21^ However, the impact on usage of novel therapies in NSCLC has not yet been characterised.

A retrospective population-based cohort study was, therefore, undertaken using updated English data from 2017 to 2021. This study aimed to describe the associations between markers of sociodemographic characteristics and novel anticancer therapy utilisation for a stage IV NSCLC population in England overall, and for the pre-pandemic and peri-pandemic periods.

## Materials and methods

### Study design and setting

A retrospective cohort was derived from data on all stage IV NSCLC (International Classification of Diseases (ICD)-10 C34.0-C34.9) diagnosed between 1 January 2017 and 31 December 2021. This period was selected due to data being considered complete and to build on previous work by Norris *et al*.^13^ This programme of work obtained favourable ethical approval from the Proportionate Review Sub-committee of the West Midlands-Edgbaston Research Ethics Committee on 16 October 2019 (REF19/WM/0317). The work is reported according to the Strengthening the Reporting of Observational Studies in Epidemiology (STROBE) guidelines (online supplemental table 2).^22^

### Data sources

All people in England diagnosed with malignant and premalignant neoplasms since 1971 are documented on the National Cancer Registration Dataset (NCRD). This is compiled by an event-based registration model from National Health Service (NHS) hospitals for analysis and release through NHS Digital.^23 24^ The following data items were accessed: quintile of Index of Multiple Deprivation (IMD) of area of residence at diagnosis as a socioeconomic status proxy^25^; sex; age; year of diagnosis; ethnicity; rural/urban category of area of residence^26^; Tumour, Node, Metastasis (TNM) summary stage at diagnosis; tumour morphology classification (ICD-02 and ICD-03)^27^; and number of comorbidities. The latter was based on the Charlson Co-morbidity Index^28^ excluding the cancer diagnosis itself, and derived from inpatient hospital admissions for the period 78 to 6 months prior to the cancer diagnosis.

The NCRD records were linked via unique patient identification numbers to the Systemic Anti-Cancer Therapy (SACT) database—a national resource recording drug-level information on cancer therapies administered by NHS providers.^29^ Established in 2012, it became mandatory to submit use of all SACT data in April 2014.

### Study population

Patients diagnosed with histologically confirmed stage IV NSCLC (adenocarcinomas, squamous cell tumours, large cell tumours, those ‘not otherwise specified’) comprised the cohort of interest. With the aim of establishing only one record per patient, a hierarchy was used to determine (in the event of a patient having multiple registrations) which tumour was retained for analysis. Stage was considered as the last step in order to retain the patient record with the most advanced lung cancer diagnosis, as some patients may have been diagnosed with two primary lung cancers of different stages across the time period of this study. It is also possible that the dataset could include two registrations of stage IV disease for the same patient if they had been diagnosed at one hospital and received a novel therapy at another. This hierarchy was as follows: (1) stages I and above only (stage 0 dropped); (2) the earliest diagnosed tumour; (3) the most advanced stage at diagnosis; (4) the most specific ICD-coded tumour (ICD-10 C34.0-C34.8); (5) the most complete data variables; and (6) the first tumour record. SCLC tumour morphology codes or ‘other specified or unspecified’ morphology codes (n=50 024); diagnosis years other than 2017–2021 (n=164 169); and stages 0–III (or stage unknown) (n=94 152) were excluded from the remaining records. This resulted in 73 640 patients for analysis.

### Outcome variable

The primary outcome of interest was utilisation of a novel anticancer therapy for stage IV NSCLC. These novel anticancer therapies included treatment with any of: (1) molecular targeted therapy; (2) biological therapy (eg, bevacizumab); (3) ICI; or (4) other novel anticancer therapy not included in other classes (eg, drug in pre-clinical development; first in class drugs; or a clinical trial in which irrespective of randomisation a novel therapy was received). While these are a diverse range of drugs, they were combined in the primary analysis to provide an overall picture of utilisation and because the percentages of patients using any specific drug were low. Chemotherapy given for NSCLC was identified (but not included as a novel anticancer therapy). Oncology expertise and available resources (eg, National Institute for Health and Care Excellence (NICE) Guidance and local SACT protocols) were used to identify and categorise drugs likely to have been used for NSCLC. A time frame restriction of 56 days before and 2 years post-diagnosis was applied to allow for the assumption that therapies of interest were prescribed for NSCLC. This time frame was selected on consensus clinician discussion to allow for a margin of error for therapies which were initiated close to the time of diagnosis, at which time the NSCLC may not have been formally coded on NHS and so registry systems; it therefore ensures such patients were not inadvertently misclassified as not having received these therapies for NSCLC. Therapies that had no indication in NSCLC were excluded from the analysis and were presumed to be for another malignancy. Drugs included in the analysis are listed in online supplemental table 1.

### Explanatory variables

Explanatory variables were related to various sociodemographic characteristics. Deprivation—a socioeconomic status proxy^25^—was estimated by IMD quintile. Quintile 1 was classified as the least deprived and, quintile 5, most deprived. Updated in 2019, this represented the IMD data closest to date of diagnosis for this cohort.^25^ Age at diagnosis was grouped as <50, 50 to 59, 60 to 69, 70 to 79, 80 to 89 and ≥90 years old in descriptive analysis; this was collapsed to 10-year intervals for multivariable logistic regression models. Ethnicity was grouped by The Office for National Statistics UK Government census list of ethnic groups as follows: ‘Asian or Asian British’; ‘Black, Black British, Caribbean or African’; ‘Mixed or Multiple Ethnic Groups’; ‘White’; ‘Other Not Otherwise Specified (NOS)’; and missing or unknown ethnicity classifications were classified as ‘Unknown’.^30^ For drug subcategory analyses, these were collapsed to ‘White’, ‘Other ethnic group’ and ‘Unknown’. Residence was categorised as ‘Rural village, hamlet, and isolated dwellings’; ‘Rural town and fringe’; ‘Urban city and town’; and ‘Urban conurbation’ (minor and major)’. These were collapsed for regression analysis to ‘Rural’ and ‘Urban’ respectively. TNM stage at diagnosis was grouped as I, II, III, IV and unknown (stage unknown or unstageable tumours). Number of comorbidities was categorised as 0, 1–2 and ≥3 for analysis.

### Statistical analysis

Descriptive demographics and clinical characteristics were reported for all patients in the cohort. The objective of the analysis was to describe the association between markers of sociodemographic characteristics and utilisation; that is, to determine whether there are any associations and, if so, estimate the direction and magnitude of these. χ² omnibus tests determined associations between demographic and clinical characteristics and novel therapy utilisation. Univariable and multivariable logistic regression models were used to estimate associations between sociodemographic characteristics and novel anticancer therapy utilisation. The focus of the multivariable model was to determine whether each of the five sociodemographic variables (age, sex, IMD, ethnicity and residence) was associated with utilisation in the presence of the others; therefore, they were all fitted in the model. Presence of comorbid conditions may preclude the use of novel therapies and has been associated with utilisation in past work (13). Because of this, and the impact of the pandemic on cancer services, the multivariable model therefore included both comorbidities and diagnostic year. ORs and 95% CIs reported the likelihood of treatment utilisation by each variable. The Hosmer and Lemeshow test was used to assess model goodness-of-fit; reported models had adequate fit.

The primary analysis related to all years, and all novel anticancer therapies combined. Exploratory secondary analyses were completed to determine associations between sociodemographic variables and utilisation (i) during the pre-COVID-19 (2017–2019) and peri-COVID-19 (2020–2021) time periods and (ii) of different drug classes. All statistical analyses were conducted using Stata version 19.0 (StataCorp., College Station, TX).

## Results

### Patient characteristics

The analysis included 73 640 patients diagnosed between 1 January 2017 and 31 December 2021. The majority of patients were aged between 60 and 89 years old (n=61 304, 83.2%) and from White ethnic groups (n=66 826, 90.7%). IMD1 quintile (most deprived) was the modal socioeconomic group (n=18 659, 25.3%). Full demographic characteristics are contained in table 1.

**Table 1:** Demographic and clinical characteristics of cohort with primary invasive stage IV NSCLC diagnosed between 1 January 2017 and 31 December 2021 (n=73 640)

Characteristic	N (%)
Sex	
Male	39 854 (54.1)
Female	33 786 (45.9)
Age (years)	
<50	2044 (2.8)
50–59	7072 (9.6)
60–69	17 536 (23.8)
70–79	26 904 (36.5)
80–89	16 864 (22.9)
≥90	3220 (4.4)
Index of Multiple Deprivation (IMD)	
1 (most deprived)	18 659 (25.3)
2	15 525 (21.1)
3	14 560 (19.8)
4	13 459 (18.3)
5 (least deprived)	11 437 (15.5)
Diagnosis year	
2017	15 328 (20.8)
2018	15 198 (20.6)
2019	14 144 (19.2)
2020	14 516 (19.7)
2021	14 454 (19.6)
Ethnicity	
Asian or Asian British	1422 (1.9)
Black, Black British, Caribbean or African	929 (1.3)
Mixed or multiple ethnic groups	278 (0.4)
White	66 826 (90.7)
Other NOS	853 (1.2)
Missing/unknown	3332 (4.5)
Rural/urban residence	
Rural village, hamlet and isolated dwellings	6064 (8.6)
Rural town and fringe	7578 (10.8)
Urban city and town	33 570 (47.7)
Urban conurbation	23 208 (33.0)
Tumour morphology	
NSCLC NOS	30 066 (40.8)
NSCLC adenocarcinoma	32 468 (44.1)
NSCLC squamous	10 370 (14.1)
NSCLC large cell	736 (1.0)
Multiple tumours	
1 tumour only	61 952 (84.1)
>1 tumour	11 688 (15.9)
Number of co-morbidities (between 78 and 6 months before diagnosis)	
0	42 229 (57.3)
1–2	21 117 (28.7)
≥3	10 294 (14.0)
SACT record in range (56 days before and 2 years after diagnosis)	
Yes (in time range)	20 589 (28.0)
Yes (not in time range)	3834 (5.21)
No	53 051 (72.0)
Treatment used	
Chemotherapy	10 517 (14.3)
Novel anticancer therapy (any)	5765 (7.8)
Targeted therapy	1542 (2.1)
Biological	671 (0.9)
ICI	4082 (5.5)
Novel NOS	83 (0.1)

ICI, immune checkpoint inhibitor; IMD, Index of Multiple Deprivation; NOS, not otherwise specified; NSCLC, non-small cell lung cancer; SACT, systemic anticancer therapy.

### Any novel anticancer therapy utilisation, 2017–2021

During 2017–2021, 5765 (7.8%) of patients diagnosed with stage IV NSCLC received a novel therapy up to 2 years post-diagnosis. Percentage utilisation was higher in females than males (n=2767, 8.2% vs n=2998, 7.5%); this persisted in adjusted analysis (multivariable OR (mvOR): 1.08, CI 1.03 to 1.15) (table 2). Percentage utilisation decreased consistently with increasing age, from 9.6% in patients <60, to 6.7% in those ≥80 years old (mvOR: 0.70, CI 0.64 to 0.77). All groups aged 70 and older had a lower likelihood of receiving a novel therapy. Utilisation by IMD quintile varied; when compared with IMD 1 (most deprived), all other quintiles had 13%–15% higher likelihood of novel therapy receipt. Variation was observed between ethnicities, with patients with ethnicity recorded as ‘Other’ and ‘Unknown’ having a 15% lower likelihood of receipt (mvOR: 0.85, CI 0.75 to 0.97). Residence (rural vs urban) did not show variation in novel therapies receipt (mvOR: 1.01, CI 0.94 to 1.08).

**Table 2:** Likelihood of using any novel anticancer therapy: univariable and multivariable ORs, 95% CIs and p values adjusted for sex, age, Index of Multiple Deprivation (IMD), diagnosis year, rural/urban residence, comorbidities and ethnicity. Statistically significant values are highlighted in bold.

Characteristic	Number (%) using a novel therapy	Number (%) not using a novel therapy	P value*	Univariable			Multivariable†		
	n=6545 (9.3%)	n=63 875 (90.7%)		OR	95% CI	P value‡	OR	95% CI	P value
Sex			**0.001**						<0.001
Male	2998 (7.5)	36 856 (92.5)		1.00	–	–	1.00	–	–
Female	2767 (8.2)	31 019 (91.8)		1.10	1.04 to 1.16	**0.001**	1.08	1.03 to 1.15	**0.004**
Age (years)			**<0.001**						<0.001
<60	876 (9.6)	8240 (90.4)		1.00	–	–	1.00	–	–
60–69	1542 (8.8)	15 994 (91.2)		0.91	0.83 to 0.99	**0.028**	0.92	0.84 to 1.01	0.066
70–79	2010 (7.5)	24 894 (92.5)		0.76	0.70 to 0.83	**<0.001**	0.79	0.73 to 0.86	**<0.001**
≥80	1337 (6.7)	18 747 (93.3)		0.67	0.61 to 0.73	**<0.001**	0.70	0.64 to 0.77	**<0.001**
Index of Multiple Deprivation (IMD)			**0.017**						<0.001
1 (most deprived)	1354 (7.3)	17 305 (92.7)		1.00	–	–	1.00	–	–
2	1258 (8.1)	14 267 (91.9)		1.13	1.04 to 1.22	**0.003**	1.15	1.05 to 1.24	**0.001**
3	1146 (7.9)	13 414 (92.1)		1.09	1.01 to 1.19	**0.035**	1.13	1.03 to 1.23	**0.006**
4	1088 (8.1)	12 371 (91.9)		1.12	1.03 to 1.22	**0.006**	1.15	1.06 to 1.26	**0.001**
5 (least deprived)	919 (8.0)	10 518 (92.0)		1.12	1.02 to 1.22	**0.013**	1.15	1.05 to 1.25	**0.004**
Diagnosis year			**<0.001**						<0.001
2017	1207 (7.9)	14 121 (92.1)		1.00	–	–	1.00	–	–
2018	1312 (8.6)	13 886 (91.4)		1.11	1.02 to 1.20	**0.016**	1.11	1.02 to 1.20	**0.018**
2019	1241 (8.8)	12 903 (91.2)		1.13	1.03 to 1.22	**0.005**	1.13	1.04 to 1.23	**0.005**
2020	1047 (7.2)	13 469 (92.8)		0.91	0.83 to 0.99	**0.031**	0.91	0.83 to 0.99	**0.036**
2021	958 (6.6)	13 496 (93.4)		0.83	0.76 to 0.91	**<0.001**	0.82	0.75 to 0.90	**<0.001**
Rural/urban residence			0.74						
Rural	1083 (7.9)	12 559 (92.1)		1.00	–	–	1.00	–	–
Urban	4459 (7.9)	52 319 (92.2)		0.99	0.92 to 1.06	0.74	1.01	0.94 to 1.08	0.864
Number of co-morbidities§			**<0.001**						<0.001
0	3548 (8.4)	38 681 (91.6)		1.00	–	–	1.00	–	–
1–2	1546 (7.3)	19 571 (92.7)		0.86	0.81 to 0.92	**<0.001**	0.90	0.85 to 0.96	**0.002**
≥3	671 (6.5)	9623 (93.5)		0.76	0.70 to 0.83	**<0.001**	0.81	0.74 to 0.89	**<0.001**
Ethnicity									0.118
White	5231 (7.8)	61 595 (92.2)	0.586	1	–	–	1	–	–
Asian or Asian British	118 (8.3)	1304 (91.7)		1.07	0.88 to 1.29	0.514	1.04	0.85 to 1.26	0.723
Black, Black British, Caribbean or African	79 (8.5)	850 (91.5)		1.09	0.87 to 1.38	0.447	1.04	0.82 to 1.32	0.755
Mixed or multiple ethnic groups	26 (9.4)	252 (90.7)		1.21	0.81 to 1.82	0.346	1.17	0.78 to 1.76	0.447
Other not otherwise specified (NOS)/unknown	311 (7.4)	37 874 (92.5)		0.95	0.84 to 1.06	0.354	0.85	0.75 to 0.97	**0.013**

* χ² p value.

† ORs are mutually adjusted for sex, age, IMD, diagnosis year, rural / urban residence, comorbidities and ethnicity.

‡ First listed p value in each row is from likelihood-ratio test (LRT) of the variable’s contribution to the model. Subsequent p values are from a test of whether the OR is different from 1.

§ Between 78 and 6 months before diagnosis.

Patients with ‘1-2’ or ‘3 or more’ co-morbidities had a 10% and 19% lower likelihood of novel therapy receipt respectively, compared with those with no comorbidities (mvOR: 0.90, CI 0.85 to 0.96 and mvOR: 0.81, CI 0.74 to 0.89 respectively). Year of diagnosis had a significant impact on utilisation; when compared with 2017, both 2018 and 2019 demonstrated increased likelihood of novel therapies receipt (mvOR: 1.11, CI 1.02 to 1.20 and mvOR: 1.13, CI 1.04 to 1.23 respectively). This changed in 2020 and 2021, with decreased likelihood of receipt observed (mvOR: 0.91, CI 0.83 to 0.99 and mvOR: 0.82, CI 0.75 to 0.90 respectively).

### Exploratory secondary analysis: utilisation pre-COVID-19 and peri-COVID-19 (2017 to 2019 and 2020 to 2021)

Of the 42 718 patients diagnosed in the pre-COVID-19 period (2017–2019), 3760 (8.4%) received a novel therapy. The patterns of variation observed in the whole cohort persisted in this sub-population. Specifically, females were more likely to receive treatment than males (mvOR: 1.09, CI 1.02 to 1.17); there was lower utilisation in those aged ≥60 compared with those <60 years old; and higher utilisation in all IMD quintiles compared with the most deprived (figure 1A). Patients whose ethnicity was documented as ‘Other’ or ‘Unknown’ again demonstrated a markedly lower likelihood of receipt (mvOR: 0.78, CI 0.67 to 0.91).

**Figure 1:**
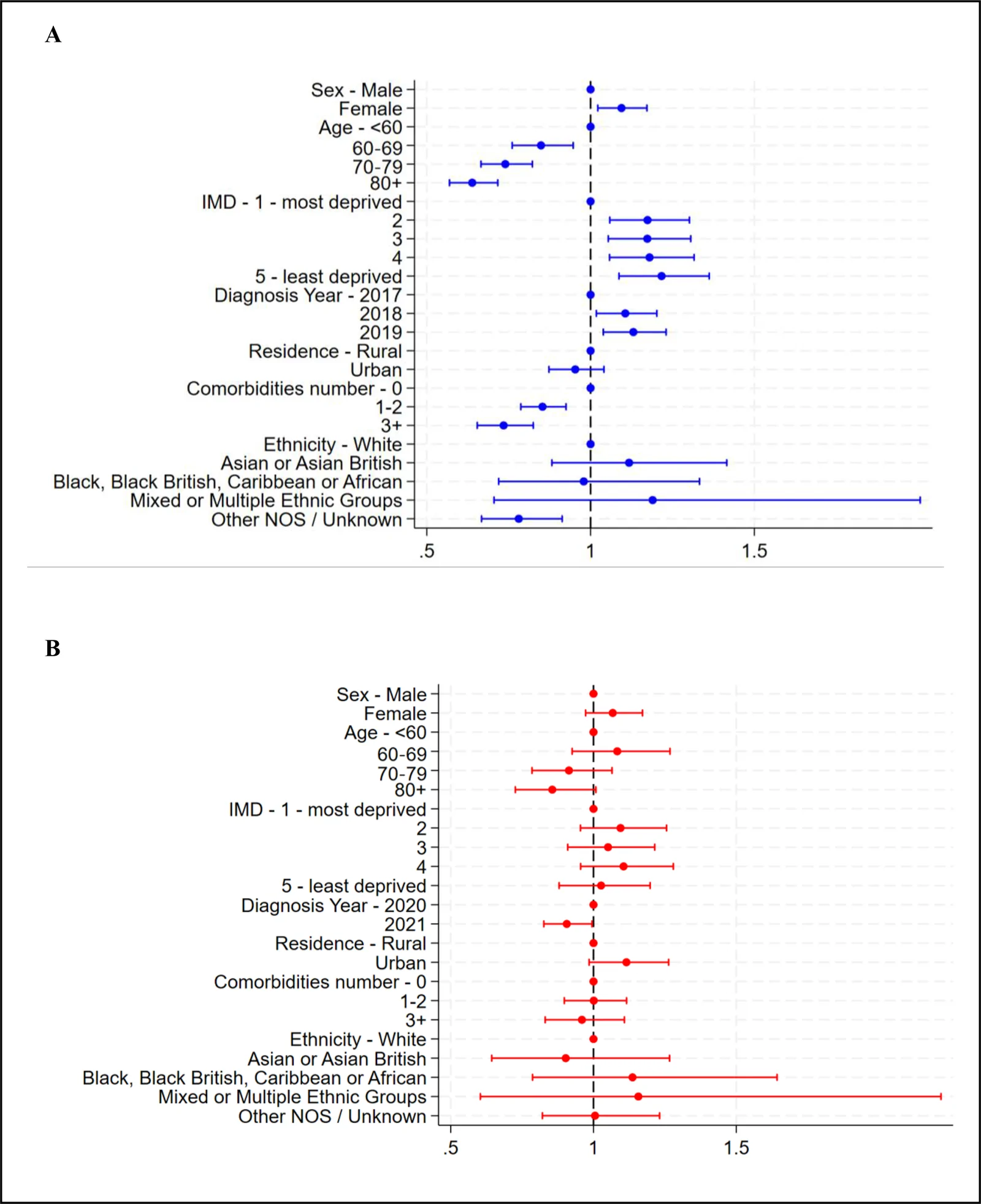
Pre-COVID-19 and peri-COVID-19 (years 2017 to 2019 (**A**); and 2020 and 2021(**B**)) forest plots of likelihood (ORs, 95% CIs and p values) of using any novel anticancer therapy adjusted for sex, age, Index of Multiple Deprivation (IMD), diagnosis year, rural/urban residence, comorbidities and ethnicity. NOS, not otherwise specified.

The peri-COVID-19 analysis included 27 702 patients diagnosed 2020 and 2021. During this time period, 2005 (6.9%) received a novel therapy. No differences were observed between sexes, age groups, IMD quintiles, co-morbidity statuses or ethnicities in the adjusted analysis (figure 1B). An almost 10% decreased likelihood of receiving a novel therapy in 2021, as opposed to 2020, was observed (mvOR: 0.91, CI 0.83 to 0.99).

### Exploratory secondary analysis: utilisation by novel drug category, 2017–2021

Of the entire study cohort, 1542 (2.1%) received a targeted therapy; 671 (0.9%) a biological therapy; 4082 (5.5%) an ICI; and 83 patients (0.1%) another novel therapy not otherwise specified.

Variation was observed in utilisation of drug categories by sociodemographic factors with ‘Other ethnic group’ being more likely to receive a targeted therapy when compared with ‘White’ and ‘Unknown’ ethnicities, but less likely to receive an ICI when compared with ‘White’ and ‘Unknown’ ethnicities (online supplemental table 5). Patients with ‘Unknown’ ethnicity were less likely to receive a biological therapy (mvOR: 0.54, CI 0.33 to 0.87) or an ICI (mvOR: 0.83, CI 0.70 to 0.98) when compared with ‘White’ ethnicity (online supplemental tables 4 and 5). Female patients were almost 20% more likely to receive targeted therapies (mvOR: 1.19, CI 1.08 to 1.32) when compared with male patients (online supplemental table 3). Across all drug categories, patients ≥70 years old were less likely to receive treatment than younger patients. Across all novel drug categories, patients in the most deprived quintile were overall less likely to receive treatment than those resident in other quintiles. The use of targeted therapies was less likely in patients diagnosed in 2020 and 2021 (mvOR: 0.75, CI 0.64 to 0.89 and mvOR: 0.66, CI 0.55 to 0.78, respectively) than in 2017 (online supplemental table 3).

## Discussion

This study represents the most up to date—and comprehensive—insight into the utilisation of novel therapies in NSCLC in the UK. This is an area in which socioeconomic inequalities have been previously demonstrated to exist^13^ and represents a disease area of unmet need with inequitable access to novel therapies.

Our findings highlight sociodemographic inequalities in the receipt of novel therapies in a national patient cohort. Those of female sex were more likely to receive novel therapies than males. Despite lung cancer being a male predominant condition, female patients are more likely to harbour actionable mutations, such as epidermal growth factor receptor (EGFR) and anaplastic lymphoma kinase (ALK).^31 32^ Patients with these driver mutations can receive targeted therapies, such as osimertinib and alectinib or brigatinib respectively^33–35^ and this likely explains our findings. Variation was observed in ethnicity and novel therapies prescribed. Those in the ‘Other ethnic group’—which contain patients from Asian backgrounds as well as others—were also more likely to receive targeted therapies. It is recognised that patients from Asian ethnic backgrounds are more likely to also have EGFR and ALK as driver mutations in their NSCLC.^32^ Investigating inequalities between ethnic groups in routine health service data, in which ethnicity data are often incomplete or lack detail, presents challenges. In this study, the lowest percentage of utilisation by ethnicity was observed to be patients whose ethnicity was recorded as ‘Other’ or ‘Unknown’. This group may contain White patients for whom ethnicity data have not been documented; however, it is likely to also contain patients from other non-majority ethnic backgrounds who are increasingly recognised to be systemically disadvantaged in healthcare services. However, in the absence of detailed ethnicity data, it is impossible to know towards which ethnic groups interventions to improve equity in access to therapies need to be targeted. This clearly demonstrates the importance of rigorous, robust and complete documentation of this information across the health service.

Further variation in prescribing was identified across deprivation quintiles, with all other IMD groups more likely to receive a novel therapy when compared with the most deprived. Lung cancer is more often diagnosed in patients from deprived socioeconomic backgrounds, and once diagnosed, these patients are more likely to die from their disease.^36^ Our findings partially reflect those of Norris *et al* for the period 2012–2017,^13^ in that they demonstrate socioeconomic inequalities in utilisation. However, we did not see the clear gradient of reducing likelihood of use with increased deprivation previously identified. While some variation existed across drug subcategory analysis, this finding was generally replicated in novel therapies. Notably, this pattern was not seen for long-established cytotoxic chemotherapy, which showed no notable variation across IMD quintiles (online supplemental table 6). From this, it may be hoped that socioeconomic inequalities in novel therapies prescribing could follow a trajectory of narrowing over time, as a result of increasing familiarity among clinicians with these drugs. This phenomenon of novel health interventions initially favouring more socioeconomically privileged members of society, and a lag being noticed in reaching those more deprived, has been observed across healthcare interventions and referred to as the ‘inverse equity hypothesis’.^37^ Prescribing of novel therapies also declined with increasing age for those aged ≥70 years old. This was observed in models which accounted for co-morbidities; and patients in this age category made up over half of the cohort (63.8%). Although this may not be considered surprising in novel drug utilisation analyses, it highlights the importance of considering equitable care, inclusive of age.

Crucially, a drop off in likelihood of utilisation of novel therapies was observed in 2020 and 2021. This change coincided with a fall in the number of lung cancer cases diagnosed,^38^ driven by the COVID-19 pandemic. Indeed, it is recognised that COVID-19 resulted in significant inequalities across health outcomes, many of which were underpinned by the fact that patients from Black, Asian and minority ethnic groups in the UK had a higher age-standardised mortality from COVID-19 than White patients.^39^ Here, what had been a trend of increasing utilisation of novel therapies in 2018 and 2019 reversed entirely. This was likely driven by interruption to normal service pathways and an increased sense of uncertainty surrounding management of non-COVID-19 conditions. Biological therapies demonstrated little variation in usage; this may be accounted for by combination therapies, using bevacizumab, being increasingly used across the later years of the study.

Another striking observation relating to the peri-pandemic period was the lack of variation in utilisation of novel therapies between sociodemographic groups. Although this may be explained in part by smaller numbers and low power to detect variation, there are points of interest in practice at this time which may have reduced socioeconomic disparities. Prescribing practices for novel therapies at this time underwent rapid changes—with more patients being reviewed by telephone; variation in terms of what was available to prescribe with interim SACT measures; and possibly a different demographic mix of patients reaching the oncology clinic, which may explain this. It will be important to monitor data for post-pandemic years—as they become available—to determine whether sociodemographic inequalities have been eliminated or re-emerge.

This study had a number of limitations which, while in many cases were unavoidable, must be acknowledged. In linking the SACT and NCRD data, it was recognised that many patients received anticancer treatments which were not prescribed for lung malignancies. Therefore, all therapies were classified as to whether they were likely to have been used for NSCLC. This resulted in a comprehensive list, accurate at time of publication, of therapies for NSCLC used in the UK between 2017 and 2021. Although this process was rigorous compared with those methods used in previous publications, it may have resulted in lower observed SACT prescribing for NSCLC than might have been expected. Limitations with the Charlson Comorbidity Index in assessing fitness for treatment with regards to age and IMD, are also recognised.

In therapy specific analysis, ethnicity categories were collapsed to ‘White’, ‘Other ethnic group’ and ‘Unknown’ to avoid having very small sub-groups in the analysis. This clearly represents an oversimplification of ethnic variation in the UK. The scope of this study was limited to patients in England due to availability of data resources. Future work should include consideration of other UK nations where patient demographic mix and local challenges of access are likely to vary and should be characterised. Furthermore, although demonstrated in trials to provide survival benefits to patient groups with NSCLC, the real-world survival of patients using novel therapies in the UK has not been comprehensively explored. Such novel therapies have also come through a clinical trials process which often recruits patients not representative of the populations the drugs will go on to be used in. Such limitations in clinical trial design and publication make real-world evidence, such as registry cohort studies, even more important in understanding how these drugs are used in our patient settings.

In conclusion, this study demonstrates there are sociodemographic inequalities in the utilisation of novel therapies for stage IV NSCLC in a publicly funded healthcare system. Next steps should include identifying the present landscape of novel therapy prescribing in the UK, and prioritising actions to address inequalities where they exist. This highlights the value of real-world cohort studies in advancing understanding of how, and in whom, therapies are used as we move into the increasingly more complicated world of precision oncology.

## Supplementary material

10.1136/jech-2025-225545 online supplemental file 1

## Data Availability

All data relevant to the study are included in the article or uploaded as supplementary information.
