# Supplementary material for: Sociodemographic inequalities in non-small cell lung cancer (NSCLC) novel treatments: a national population-based cohort study
Source: J Epidemiol Community Health. 2026 Jun 30;80(11):e225545. doi: 10.1136/jech-2025-225545 (PMC13691723; doi:10.1136/jech-2025-225545)
Supplement: Supplementary file 1 [file jech-80-11-s001.pdf]

Supplement: Sociodemographic Inequalities in Non-Small Cell Lung Cancer (NSCLC) Novel Treatments: A Population-Based Cohort Study

<u>Supplementary Table 1 | Drugs categorisation Table</u>

SACT Drug Classification	Individual Drug	Combination Therapy
**Targeted Therapy**	AFATINIB<br>ALECTINIB<br>BRIGATINIB<br>CERITINIB<br>COBIMETINIB<br>CRIZOTINIB<br>PRALSETINIB<br>DABRAFENIB<br>DACOMITINIB<br>ENCORAFENIB<br>ENTRECTINIB<br>REPOTRECTINIB<br>ERLOTINIB<br>GEFITINIB<br>LENVATINIB<br>LORLATINIB<br>OSIMERTINIB<br>PALBOCICLIB<br>SOTORASIB<br>TEPOTINIB<br>TRAMETINIB<br>VEMURAFENIB<br>MOBOCERTINIB<br>SELPERCATINIB	DABRAFENIB + TRAMETINIB
**Targeted Therapy + ICI Combination**		LENVATINIB + PEMBROLIZUMAB
**Biological Therapy**	BEVACIZUMAB<br>BEVACIZUMAB (AVASTIN)<br>BEVACIZUMAB BIOSIMILAR (ALYMSYS)<br>BEVACIZUMAB BIOSIMILAR (OYAVAS)<br>NINTEDANIB<br>RAMUCIRUMAB	BEVACIZUMAB + CARBO<br>BEVACIZUMAB + CARBOPLATIN<br>BEVACIZUMAB + CARBO + PACLITAXEL<br>BEVACIZUMAB + CARBOPLATIN + PACLITAXEL<br>BEVACIZUMAB + CISPLATIN + PACLITAXEL<br>BEVACIZUMAB + PACLITAXEL<br>BEVACIZUMAB + CARBOPLATIN + DOCETAXEL<br>DOCETAXEL + NINTEDANIB
**Biological Therapy + ICI Combination**		ATEZOLIZUMAB + BEVACIZUMAB + CARBOPLATIN + PACLITAXEL<br>ATEZOLIZUMAB + BEVACIZUMAB + CARBOPLATIN + PACLITAXEL
**Chemotherapy + Biological Therapy + ICI Combination**		ATEZOLIZUMAB + BEVACIZUMAB
**Immune checkpoint inhibitor (ICI)**	ATEZOLIZUMAB<br>AVELUMAB<br>CEMIPLIMAB<br>DOSTARLIMAB<br>DURVALUMAB<br>IPILIMUMAB<br>NIVOLUMAB	CARBOPLATIN + PACLITAXEL + PEMBROLIZUMAB<br>CARBOPLATIN + PEMBROLIZUMAB + PEMETREXED

	PDR001<br>PEMBROLIZUMAB<br>TREMELIMUMAB<br>IPILIMUMAB + NIVOLUMAB<br>REFINE-LUNG TRIAL	CISPLATIN +<br>PEMBROLIZUMAB +<br>PEMETREXED<br>PEMBROLIZUMAB +<br>PEMETREXED
**Utilised Targeted Therapy + ICI Combination**		LENVATINIB +<br>PEMBROLIZUMAB
**Biological Therapy + ICI Combination**		ATEZOLIZUMAB +<br>BEVACIZUMAB
**Chemotherapy + Biological Therapy + ICI Combination**		ATEZOLIZUMAB +<br>BEVACIZUMAB +<br>CARBOPLATIN +<br>PACLITAXEL<br>ATEZOLIZUMAB +<br>BEVACIZUMAB +<br>CARBOPLATIN +<br>PACLITAXEL
**Chemotherapy**	CARBOPLATIN<br>CISPLATIN<br>DOCETAXEL<br>GEMCITABINE<br>PACLITAXEL<br>PEMETREXED<br>VINORELBINE<br>CAPITAL TRIAL<br>DOCETAXEL<br>NAB-PACLITAXEL	CARBOPLATIN +<br>PACLITAXEL<br>CARBOPLATIN +<br>GEMCITABINE<br>CARBOPLATIN +<br>PACLITAXEL + RT<br>CARBOPLATIN +<br>PEMETREXED<br>CARBOPLATIN + RT<br>CARBOPLATIN +<br>VINORELBINE CARBOPLATIN<br>+ VINORELBINE + RT<br>CISPLATIN + DOCETAXEL<br>CISPLATIN + ETOPOSIDE<br>CISPLATIN + ETOPOSIDE +<br>RT<br>CISPLATIN + GEMCITABINE<br>CISPLATIN + PACLITAXEL<br>CISPLATIN + PEMETREXED<br>CISPLATIN + RT<br>CISPLATIN + VINORELBINE<br>CISPLATIN + VINORELBINE +<br>RT<br>GEMCARBO<br>GEMCITABINE + RT<br>GEMCITABINE +<br>VINORELBINE<br>WT SIOP 2002 01 POST OP<br>CARBO, ETOP<br>CARBOPLATIN + NAB-<br>PACLITAXEL<br>CISPLATIN + MITOMYCIN +<br>VINBLASTINE
**Novel not otherwise specified**	SACITUZUMAB GOVITECAN<br>TRASTUZUMAB<br>DERUXTECAN<br>MATRIX LUNG TRIAL	

<u>Supplementary Table 2 | STROBE Checklist</u>

STROBE Statement—Checklist of items that should be included in reports of ***cohort studies***

	Item No	Recommendation	Page No
**Title and abstract**	1	(*a*) Indicate the study's design with a commonly used term in the title or the abstract<br>(*b*) Provide in the abstract an informative and balanced summary of what was done and what was found	1,3
**Introduction**			
Background/rationale	2	Explain the scientific background and rationale for the investigation being reported	6,7
Objectives	3	State specific objectives, including any prespecified hypotheses	7
**Methods**			
Study design	4	Present key elements of study design early in the paper	8-10
Setting	5	Describe the setting, locations, and relevant dates, including periods of recruitment, exposure, follow-up, and data collection	8
Participants	6	(*a*) Give the eligibility criteria, and the sources and methods of selection of participants. Describe methods of follow-up<br>(*b*) For matched studies, give matching criteria and number of exposed and unexposed	8-10
Variables	7	Clearly define all outcomes, exposures, predictors, potential confounders, and effect modifiers. Give diagnostic criteria, if applicable	9-10
Data sources/ measurement	8*	For each variable of interest, give sources of data and details of methods of assessment (measurement). Describe comparability of assessment methods if there is more than one group	8-10
Bias	9	Describe any efforts to address potential sources of bias	8-9
Study size	10	Explain how the study size was arrived at	8
Quantitative variables	11	Explain how quantitative variables were handled in the analyses. If applicable, describe which groupings were chosen and why	8-9
Statistical methods	12	(*a*) Describe all statistical methods, including those used to control for confounding<br>(*b*) Describe any methods used to examine subgroups and interactions<br>(*c*) Explain how missing data were addressed<br>(*d*) If applicable, explain how loss to follow-up was addressed<br>(*e*) Describe any sensitivity analyses	10
**Results**			
Participants	13*	(a) Report numbers of individuals at each stage of study—eg numbers potentially eligible, examined for eligibility, confirmed eligible, included in the study, completing follow-up, and analysed<br>(b) Give reasons for non-participation at each stage<br>(c) Consider use of a flow diagram	11
Descriptive data	14*	(a) Give characteristics of study participants (eg demographic, clinical, social) and information on exposures and potential confounders<br>(b) Indicate number of participants with missing data for each variable of interest<br>(c) Summarise follow-up time (eg, average and total amount)	11-13, 21-22
Outcome data	15*	Report numbers of outcome events or summary measures over time	11-13

Main results	16	(*a*) Give unadjusted estimates and, if applicable, confounder-adjusted estimates and their precision (eg, 95% confidence interval). Make clear which confounders were adjusted for and why they were included<br>(*b*) Report category boundaries when continuous variables were categorized<br>(*c*) If relevant, consider translating estimates of relative risk into absolute risk for a meaningful time period	11-13, 23-24
Other analyses	17	Report other analyses done—eg analyses of subgroups and interactions, and sensitivity analyses	12, Supp.
**Discussion**			
Key results	18	Summarise key results with reference to study objectives	13-16
Limitations	19	Discuss limitations of the study, taking into account sources of potential bias or imprecision. Discuss both direction and magnitude of any potential bias	15
Interpretation	20	Give a cautious overall interpretation of results considering objectives, limitations, multiplicity of analyses, results from similar studies, and other relevant evidence	14-16
Generalisability	21	Discuss the generalisability (external validity) of the study results	15
**Other information**			
Funding	22	Give the source of funding and the role of the funders for the present study and, if applicable, for the original study on which the present article is based	1

*Give information separately for exposed and unexposed groups.

Note: An Explanation and Elaboration article discusses each checklist item and gives methodological background and published examples of transparent reporting. The STROBE checklist is best used in conjunction with this article (freely available on the Web sites of PLoS Medicine at http://www.plosmedicine.org/, Annals of Internal Medicine at http://www.annals.org/, and Epidemiology at http://www.epidem.com/). Information on the STROBE Initiative is available at http://www.strobe-statement.org.

Supplementary Table 3 | Likelihood of using targeted therapy: univariable and multivariable odds ratios (OR), 95% confidence intervals (CIs) and p values adjusted for sex, age, Index of Multiple Deprivation (IMD), diagnosis year, rural / urban residence and ethnicity

Characteristic	Number (%) using a targeted therapy (n = 1,542)	Number (%) not using a targeted therapy (n = 72,098)	*p* Value[a]	Univariable			Multivariable [b]		
				OR	95% CI	*p* Value[c]	OR	95% CI	*p* Value
**Sex**									0.001
Male	771 (1.9)	39,083 (98.1)	**0.001**	1.00	-	-	1.00	-	-
Female	771 (2.3)	33,015 (97.7)		1.18	1.07-1.31	**0.001**	1.19	1.08-1.32	**0.001**
**Age (years)**									<0.001
<60	241 (2.6)	8,875 (97.4)	**<0.001**	1.00	-	-	1.00	-	-
60-69	398 (2.3)	17,138 (97.7)		0.86	0.73-1.01	0.058	0.86	0.72-1.01	0.065
70-79	540 (2.0)	26,364 (98.0)		0.75	0.65-0.88	**<0.001**	0.77	0.66-0.90	**0.001**
≥80	363 (1.8)	19,721 (98.2)		0.68	0.57-0.80	**<0.001**	0.68	0.57-0.81	**<0.001**
**Index of Multiple Deprivation (IMD)**									0.018
1 (most deprived)	345 (1.9)	18,314 (98.2)	0.053	1.00	-	-	1.00	-	-
2	341 (2.2)	15,184 (97.8)		1.19	1.02-1.39	**0.023**	1.22	1.04-1.42	**0.013**
3	305 (2.1)	14,255 (97.9)		1.14	0.97-1.33	0.109	1.16	0.99-1.37	0.070
4	311 (2.3)	13,148 (97.7)		1.26	1.08-1.47	**0.004**	1.30	1.10-1.53	**0.002**
5 (least deprived)	240 (2.1)	11,197 (97.9)		1.14	0.96-1.34	0.128	1.18	0.99-1.40	0.064
**Diagnosis Year**									<0.001
2017	361 (2.4)	14,967 (97.6)	**<0.001**	1.00	-	-	1.00	-	-
2018	363 (2.4)	14,835 (97.6)		1.01	0.88-1.18	0.848	1.01	0.87-1.18	0.847
2019	328 (2.3)	13,816 (97.7)		0.98	0.85-1.15	0.837	0.98	0.84-1.15	0.809
2020	258 (1.8)	14,258 (98.2)		0.75	0.64-0.88	**<0.001**	0.75	0.64-0.89	**0.001**
2021	232 (1.6)	14,222 (98.4)		0.68	0.57-0.80	**<0.001**	0.66	0.55-0.78	**<0.001**
**Rural / urban residence**									
Rural	309 (2.3)	13,333 (97.7)	0.149	1.00	-	-	1.00	-	-

Urban	1,174 (2.1)	55,604 (97.9)		0.91	0.80-1.03	0.150	0.92	0.81-1.05	0.205
**Ethnicity**									0.003
White	1,366 (2.0)	65,460 (98.0)	**0.001**	1.00	-	-	1.00	-	-
Other ethnic group	104 (3.0)	3,378 (97.0)		1.15	1.21-1.81	**<0.001**	1.47	1.19-1.81	**<0.001**
Unknown	72 (2.2)	3,260 (97.8)		1.05	0.83-1.34	0.643	1.01	0.79-1.30	0.928

[a] Chi-square *p* value.
[b] ORs are mutually adjusted for sex, age, IMD, diagnosis year, rural / urban residence and ethnicity
[c]First listed p value in each row is from likelihood-ratio test (LRT) of the variable's contribution to the model. Subsequent p values are from a test of whether the OR is different from 1.

Supplementary Table 4 | Likelihood of using biological therapy: univariable and multivariable odds ratios (OR), 95% confidence intervals (CIs) and p values adjusted for sex, age, Index of Multiple Deprivation (IMD), diagnosis year, rural / urban residence, comorbidities and ethnicity

Characteristic	Number (%) using a biological therapy (n = 671)	Number (%) not using a biological therapy (n = 72,969)	*p* Value[a]	Univariable			Multivariable[b]		
				OR	95% CI	*p* Value[c]	OR	95% CI	*p* Value
**Sex**									0.633
Male	357 (0.9)	39,497 (99.1)	0.632	1.00	-	-	1.00	-	-
Female	314 (0.9)	33,472 (99.1)		1.04	0.89-1.21	0.632	1.04	0.89-1.22	0.601
**Age (years)**									<0.001
<60	128 (1.4)	8,988 (98.6)	**<0.001**	1.00	-	-	1.00	-	-
60-69	203 (1.2)	17,333 (98.8)		0.82	0.66-1.03	0.085	0.83	0.66-1.05	0.123
70-79	195 (0.7)	26,709 (99.3)		0.51	0.41-0.64	<0.001	0.51	0.40-0.65	**<0.001**
≥80	145 (0.7)	19,939 (99.3)		0.51	0.40-0.65	<0.001	0.53	0.41-0.68	**<0.001**
**Index of Multiple Deprivation (IMD)**									0.013
1 (most deprived)	150 (0.8)	18,509 (99.2)	**0.040**	1.00	-	-	1.00	-	-
2	138 (0.9)	15,387 (99.1)		1.11	0.88-1.40	0.392	1.16	0.91-1.47	0.236
3	152 (1.0)	14,408 (99.0)		1.30	1.04-1.63	**0.023**	1.39	1.09-1.76	**0.007**
4	141 (1.0)	13,318 (99.0)		1.31	1.04-1.65	**0.023**	1.37	1.07-1.74	**0.012**
5 (least deprived)	90 (0.7)	11,347 (99.2)		0.98	0.75-1.27	0.872	1.02	0.77-1.34	0.912
**Diagnosis Year**									0.700
2017	151 (1.0)	15,177 (99.0)	0.672	1.00	-	-	1.00	-	-
2018	138 (0.9)	15,060 (99.1)		0.92	0.73-1.16	0.487	0.92	0.73-1.17	0.500
2019	135 (1.0)	14,009 (99.1)		0.97	0.76-1.22	0.789	1.00	0.79-1.26	0.971
2020	125 (0.9)	14,391 (99.1)		0.87	0.69-1.11	0.264	0.87	0.68-1.11	0.260
2021	122 (0.8)	14,332 (99.2)		0.86	0.67-1.09	0.202	0.87	0.68-1.10	0.245

**Rural / urban residence**									
Rural	137 (1.0)	13,505 (99.0)	0.252	1.00	-	-	1.00	-	-
Urban	511 (0.9)	56,267 (99.1)		0.90	0.74-1.08	0.252	0.93	0.77-1.14	0.487
**Number of co-morbidities[d]**									0.386
0	412 (1.0)	41,817 (99.0)	**0.033**	1.00	-	-	1.00	-	-
1-2	186 (0.9)	20,931 (99.1)		0.90	0.76-1.07	0.245	0.98	0.82-1.18	0.838
≥3	73 (0.7)	10,221 (99.3)		0.72	0.56-0.93	**0.012**	0.82	0.63-1.06	0.134
**Ethnicity**									0.013
White	624 (0.9)	66,202 (99.0)	0.058	1.00	-	-	1.00	-	-
Other ethnic group	29 (0.8)	3,453 (99.2)		0.89	0.61-1.29	0.545	0.81	0.55-1.18	0.266
Unknown	18 (0.5)	3,314 (99.5)		0.58	0.36-0.92	**0.021**	0.54	0.33-0.87	**0.012**

[a] Chi-square *p* value.
[b] ORs are mutually adjusted for sex, age, IMD, diagnosis year, rural / urban residence, comorbidities and ethnicity
[c]First listed p value in each row is from likelihood-ratio test (LRT) of the variable's contribution to the model. Subsequent p values are from a test of whether the OR is different from 1.
[d] Between 78 and 6 months before diagnosis.

Supplementary Table 5 | Likelihood of using ICI therapy: univariable and multivariable odds ratios (OR), 95% confidence intervals (CIs) and p values adjusted for sex, age, Index of Multiple Deprivation (IMD), diagnosis year, rural / urban residence, comorbidities and ethnicity

Characteristic	Number (%) using an ICI (n = 4,082)	Number (%) not using an ICI (n = 69,558)	*p* Value[a]	Univariable			Multivariable[b]		
				OR	95% CI	*p* Value[c]	OR	95% CI	*p* Value
**Sex**									0.035
Male	2,114 (5.4)	37,710 (94.6)	**0.035**	1.00	-	-	1.00	-	-
Female	1,938 (5.7)	31,848 (94.3)		1.07	1.00-1.14	**0.035**	1.05	0.99-1.12	0.116
**Age (years)**									<0.001
<60	610 (6.7)	8,506 (93.3)	**<0.001**	1.00	-	-	1.00	-	-
60-69	1,098 (6.3)	16,438 (93.7)		0.93	0.84-1.03	0.174	0.95	0.85-1.05	0.326
70-79	1,435 (5.3)	25,469 (94.7)		0.79	0.71-0.87	**<0.001**	0.82	0.74-0.91	**<0.001**
≥80	939 (4.7)	19,145 (95.3)		0.68	0.62-0.76	**<0.001**	0.71	0.64-0.80	**<0.001**
**Index of Multiple Deprivation (IMD)**									0.049
1 (most deprived)	975 (5.2)	17,684 (94.8)	0.279	1.00	-	-	1.00	-	-
2	876 (5.6)	14,649 (94.4)		1.08	0.99-1.19	0.090	1.10	0.99-1.21	0.065
3	816 (5.6)	13,744 (94.4)		1.08	0.98-1.18	0.129	1.11	1.01-1.23	**0.038**
4	758 (5.6)	12,701 (94.4)		1.08	0.98-1.19	0.112	1.11	1.00-1.23	**0.049**
5 (least deprived)	657 (5.7)	10,780 (94.3)		1.11	1.00-1.22	0.054	1.13	1.02-1.26	**0.023**
**Diagnosis Year**									<0.001
2017	814 (5.3)	14,514 (94.7)	**<0.001**	1.00	-	-	1.00	-	-
2018	930 (6.1)	14,268 (93.9)		1.16	1.05-1.28	**0.002**	1.16	1.06-1.29	**0.003**
2019	881 (6.2)	13,263 (93.8)		1.18	1.07-1.31	**0.001**	1.19	1.08-1.32	**0.001**
2020	770 (5.3)	13,746 (94.7)		1.00	0.90-1.11	0.981	1.00	0.90-1.11	0.999
2021	687 (4.8)	13,767 (95.3)		0.89	0.80-0.99	**0.028**	0.89	0.80-0.98	**0.025**

**Rural / urban residence**									
Rural	744 (5.5)	12,898 (94.6)	0.0522	1.00	-	-	1.00	-	-
Urban	3,176 (5.6)	53,602 (94.4)		1.03	0.95-1.12	0.522	1.05	0.97-1.14	0.251
**Number of co-morbidities**[d]									0.0002
0	2,519 (6.0)	39,710 (94.0)	**<0.001**	1.00	-	-	1.00	-	-
1-2	1,088 (5.2)	20,029 (94.9)		0.86	0.80-0.92	**<0.001**	0.89	0.82-0.96	**0.002**
≥3	475 (4.6)	9,819 (95.4)		0.76	0.69-0.84	**<0.001**	0.81	0.73-0.91	**<0.001**
**Ethnicity**									0.011
White	3,733 (5.6)	63,093 (94.4)	0.279	1.00	-	-	1.00	-	-
Other ethnic group	179 (5.1)	3,303 (94.9)		0.92	0.79-1.07	0.264	0.85	0.73-1.00	**0.045**
Unknown	170 (5.1)	3,162 (94.6)		0.91	0.78-1.06	0.234	0.83	0.70-0.98	**0.026**

[a] Chi-square *p* value.
[b] ORs are mutually adjusted for sex, age, IMD, diagnosis year, rural / urban residence, comorbidities and ethnicity
[c]First listed p value in each row is from likelihood-ratio test (LRT) of the variable’s contribution to the model. Subsequent p values are from a test of whether the OR is different from 1.
[d] Between 78 and 6 months before diagnosis.

Supplementary Table 6 | Likelihood of using chemotherapy: univariable and multivariable odds ratios (OR), 95% confidence intervals (CIs) and p values adjusted for sex, age, Index of Multiple Deprivation (IMD), diagnosis year, rural / urban residence, comorbidities and ethnicity

Characteristic	Number (%) using chemotherapy (n = 10,517)	Number (%) not using chemotherapy (n = 63,123)	*p* Value[a]	Univariable			Multivariable[b]		
				OR	95% CI	*p* Value[c]	OR	95% CI	*p* Value
**Sex**									0.135
Male	5,621 (14.1)	34,233 (85.9)	0.135	1.00	-	-	1.00	-	-
Female	4,896 (14.5)	28,890 (85.5)		1.03	0.99-1.08	0.135	1.03	0.98-1.07	0.216
**Age (years)**									<0.001
<60	1,518 (16.7)	7,598 (83.4)	**<0.001**	1.00	-	-	1.00	-	-
60-69	2,718 (15.5)	14,818 (84.5)		0.92	0.86-0.98	**0.015**	0.93	0.87-1.00	**0.046**
70-79	3,793 (14.1)	23,111 (85.9)		0.82	0.77-0.88	**<0.001**	0.84	0.78-0.90	**<0.001**
≥80	2,488 (12.4)	17,596 (87.6)		0.71	0.66-0.76	**<0.001**	0.74	0.68-0.79	**<0.001**
**Index of Multiple Deprivation (IMD)**									0.286
1 (most deprived)	2,644 (14.2)	16,015 (85.8)	0.585	1.00	-	-	1.00	-	-
2	2,232 (14.4)	13,293 (85.6)		1.02	0.96-1.08	0.586	1.02	0.96-1.09	0.490
3	2,046 (14.1)	12,514 (86.0)		0.99	0.93-1.05	0.759	1.01	0.94-1.07	0.877
4	1,976 (14.7)	11,483 (85.3)		1.04	0.98-1.11	0.197	1.06	0.99-1.13	0.108
5 (least deprived)	1,619 (14.2)	9,818 (85.8)		1.00	0.93-1.07	0.972	1.02	0.95-1.09	0.632
**Diagnosis Year**									<0.001
2017	2,523 (16.5)	12,805 (83.5)	**<0.001**	1.00	-	-	1.00	-	-
2018	2,281 (15.0)	12,917 (85.0)		0.90	0.84-0.95	**<0.001**	0.90	0.84-0.95	**0.001**

2019	2,068 (14.6)	12,076 (85.4)		0.87	0.82-0.93	**<0.001**	0.87	0.82-0.93	**<0.001**
2020	1,876 (12.9)	12,640 (87.1)		0.75	0.71-0.80	**<0.001**	0.76	0.71-0.81	**<0.001**
2021	1,769 (12.2)	12,685 (87.8)		0.71	0.66-0.76	**<0.001**	0.70	0.66-0.75	**<0.001**
**Rural / urban residence**									
Rural	2,006 (14.7)	11,636 (85.3)	0.104	1.00	-	-	1.00	-	-
Urban	8,041 (14.2)	48,737 (85.8)		1.04	0.91-1.01	0.104	0.96	0.90-1.01	0.111
**Number of co-morbidities**[d]									<0.001
0	6,360 (15.1)	35,869 (84.9)	**<0.001**	1.00	-	-	1.00	-	-
1-2	2,886 (13.7)	18,231 (86.3)		0.89	0.85-0.94	**<0.001**	0.93	0.88-0.97	**0.003**
≥3	1,271 (12.4)	9,023 (87.7)		0.79	0.74-0.85	**<0.001**	0.84	0.79-0.90	**<0.001**
**Ethnicity**									0.004
White	9,576 (14.3)	57,250 (85.7)	0.125	1.00	-	-	1.00	-	-
Other ethnic group	505 (14.5)	2,977 (85.5)		1.01	0.92-1.12	0.776	0.96	0.87-1.06	0.409
Unknown	436 (13.1)	2,896 (86.9)		0.90	0.81-1.00	**0.045**	0.84	0.75-0.94	**0.001**

[a] Chi-square *p* value.
[b] ORs are mutually adjusted for sex, age, IMD, diagnosis year, rural / urban residence, comorbidities and ethnicity
[c]First listed p value in each row is from likelihood-ratio test (LRT) of the variable's contribution to the model. Subsequent p values are from a test of whether the OR is different from 1.
[d] Between 78 and 6 months before diagnosis.
